# Evaluation of dynamic developmental processes and the molecular basis of the high body fat percentage of different proglottid types of *Moniezia expansa*

**DOI:** 10.1186/s13071-019-3650-1

**Published:** 2019-08-05

**Authors:** Yi Liu, Zhengrong Wang, Shuai Pang, Wenjuan Zhao, Lichao Kang, Yanyan Zhang, Hui Zhang, Jingquan Yang, Zhixin Wang, Pingping Lu, Mengfei Xu, Weiyi Wang, Xinwen Bo, Zhenzhen Li

**Affiliations:** 1 State Key Laboratory of Sheep Genetic Improvement and Healthy Production/Institute of Animal Husbandry and Veterinary, Xinjiang Academy of Agricultural and Reclamation Sciences, Shihezi, China; 2 Novogene Bioinformatics Institute, Beijing, China; 3 Yangcheng Country Animal Husbandry and Veterinary Bureau, Jincheng, China; 4 Department of Medical Microbiology and Parasitology, School of Basic Medical Sciences, Fudan University, Shanghai, China; 5 Xinjiang Tiankang Feed Technology Co., Ltd, Ürümqi, China

**Keywords:** *Moniezia expansa*, RNA-seq, Lipid, Development, Proglottids, Homeobox, Wnt

## Abstract

**Background:**

*Moniezia expansa* (Cyclophyllidea: Anoplocephalidae) is a large species of tapeworm that occurs in sheep and cattle and inhabits the small intestine, causing diarrhea and weight declines, leading to stockbreeding losses. Interestingly, the body fat percentage of *M. expansa*, which lacks the ability to synthesize fatty acids, is as high as 78% (dry weight) and all of the proglottids of *M. expansa* exhibit a dynamic developmental process from top to bottom. The aim of this paper is to identify the molecular basis of this high body fat percentage, the dynamic expression of developmental genes and their expression regulation patterns.

**Results:**

From 12 different proglottids (four sections: scolex and neck, immature, mature and gravid with three replicates), 13,874 transcripts and 680 differentially expressed genes (DEGs) were obtained. The gene expression patterns of the scolex and neck and immature proglottids were very similar, while those of the mature and gravid proglottids differed greatly. In addition, 13 lipid transport-related proteins were found in the DEGs, and the expression levels showed an increasing trend in the four proglottid types. Furthermore, it was shown that 33 homeobox genes, 9 of which were DEGs, had the highest expression in the scolex and neck section. The functional enrichment results of the DEGs were predominantly indicative of development-related processes, and there were also some signal transduction and metabolism results. The most striking result was the finding of Wnt signaling pathways, which appeared multiple times. Furthermore, the weighted gene co-expression networks were divided into 12 modules, of which the brown module was enriched with many development-related genes.

**Conclusions:**

We hypothesize that *M. expansa* uses lipid transport-associated proteins to transport lipids from the host gut to obtain energy to facilitate its high fecundity. In addition, homeobox genes and Wnt signaling pathways play a core role in development and regeneration. The results promote research on the cell differentiation involved in the continuous growth and extension of body structures.

**Electronic supplementary material:**

The online version of this article (10.1186/s13071-019-3650-1) contains supplementary material, which is available to authorized users.

## Background

*Moniezia expansa* is a parasite that is commonly found in the small intestines of cattle, sheep, goats, deer, etc. [1]. It is mainly harmful to lambs and calves, while mature animals generally have no clinical symptoms. Sick lambs become thin, are cast out of the fold, and produce soft feces, which then develops into diarrhea accompanied by mucus and gestational segments. An excessive number of worms can sometimes form aggregations that cause intestinal obstruction, intestinal torsion, and even intestinal rupture. In severe cases, the animals become anemic and weak, and some even exhibit no purposeful walking, a stumbling gait, tremors and other neurological symptoms [2]. There are two hosts in the life history of *M. expansa*. The ruminant definitive hosts excrete the eggs and gravid proglottids with the feces. After the eggs are swallowed by a mite intermediate host, the oncospheres pass through the wall of the digestive tract and enter the body cavity to develop into infectious cysticerci. Finally, ruminants are infected by swallowing a mite containing the cysticerci when grazing. The cysticerci become mature after 37–40 days in lambs and after 47–50 days in calves. Tapeworms are spontaneously excreted from the body after living in an animal’s small intestine for two to six months [3].

*Moniezia expansa* (as all cestodes) lacks digestive organs, and its tegument is covered with numerous tiny finger-like specialized structures called microtriches. The structure of microtriches is similar to that of intestinal villi, except the ends are spiked. The entire tegument of the worm is covered with microtriches, including the surface of the suckers, to absorb nutrients from the intestine. The fluid in the small intestine is rich in high-, medium- and low-density lipoproteins, triglycerides, phospholipids, ether lipids, cholesterol, linoleic acid, linolenic acid and unsaturated fatty acids. Our previous research showed the body fat content of *M. expansa* was 78% (dry weight) according to the national food safety standard GB5009.6-2016 (the determination of fat use acid-hydrolysis method).However, studies in Platyhelminthes have shown that the free-living Turbellaria [4] and the parasitic Cestoda [5] and Trematoda [6] have lost the ability to synthesize fatty acids, and the broad absence of this ability in flatworms is likely to represent an ancestral loss. Therefore, why is the body fat content of *M. expansa* so high? One possible reason is that the metabolism of lipids in *M. expansa* has special features.

The body of *M. expansa* consists of three parts: the scolex, the neck and the strobila. The neck section is a very small part that occurs after the scolex section, and its function is to continuously bud off the segments. The strobila segments can be divided into immature, mature and gravid proglottids according to their anteroposterior position and the development of the sexual organs (*M. expansa* is androgynous) [7]. Each newly formed proglottid moves toward the posterior end as a new one takes its place, and during the process becomes sexually mature. The gravid or senile terminal proglottids detach or disintegrate. This process essentially involves the time-dependent expression of genes. Studying the dynamic expression of functional genes and their expression regulation patterns will identify new ways to further study the genes related to development.

As an increasing number of genomes of parasite species are sequenced, a more comprehensive perspective on some of the parasitic adaptations developed by parasites during evolution is revealed. However, most available information on development is based on traditional model organisms (*Drosophila* and *Caenorhabditis elegans*), and among Platyhelminthes, only planarians have been studied in detail, while the parasitic taxa have been largely overlooked [8]. However, parasites exhibit a complicated lifestyle (with at least one host), a capacity for unlimited growth and high fecundity, which offers unique opportunities to address long-standing questions related to development. Olson et al. [9] used *Hymenolepis microstoma*, a parasite of the bile duct of mice, as a model to study the molecular differences in the development of different segments [9]. Here, developmental genes from four different types of proglottids of *M. expansa* were investigated using RNA-seq, providing a systematic analysis of the gene expression patterns.

## Methods

### Sample collection

**Fig. 1:**
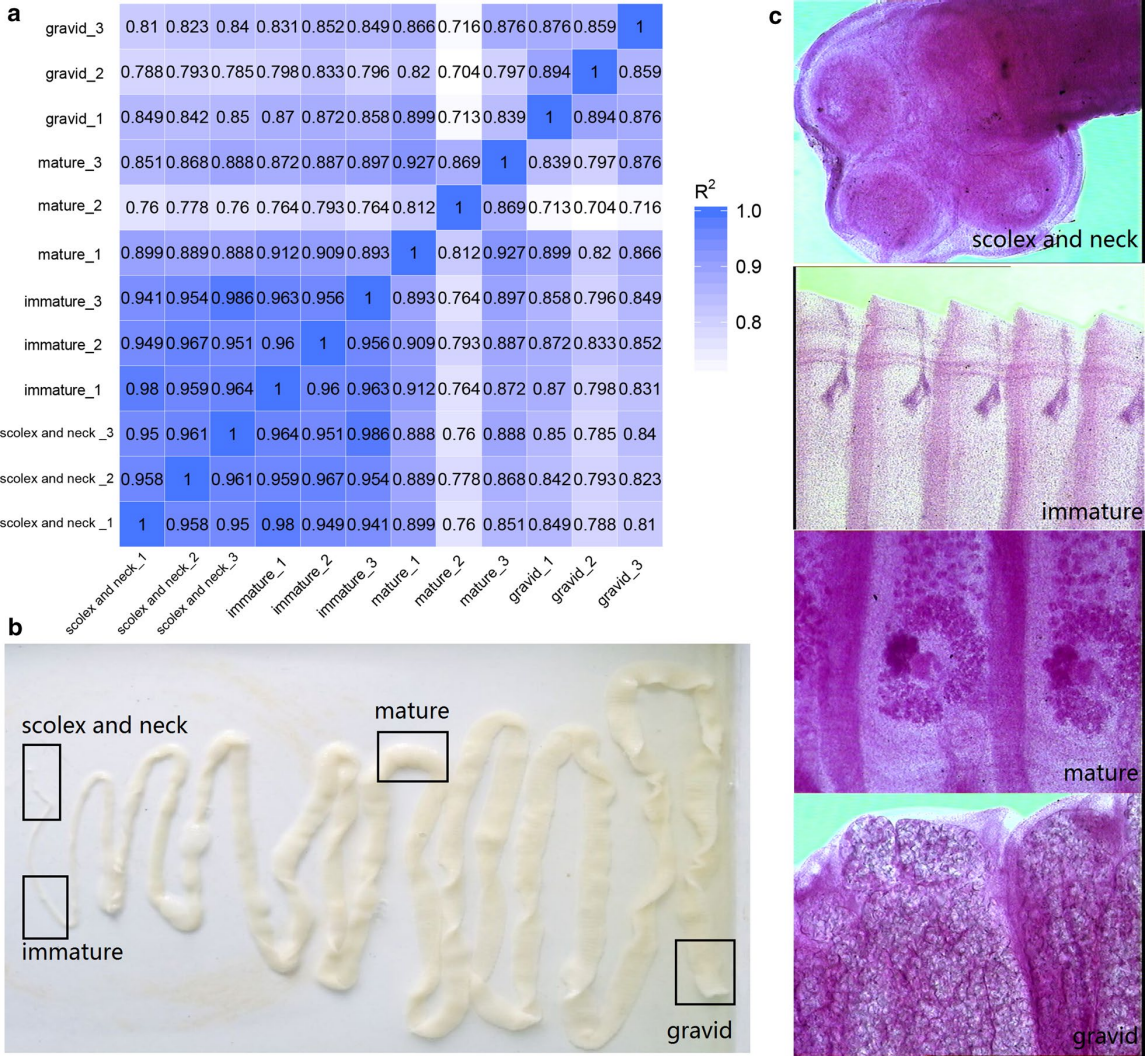
** a** Heat map: correlation coefficients among samples. *R*^2^: square of the Pearsonʼs correlation coefficient. **b** Diagrammatic representation of *M. expansa* used for RNA-seq. Boxes indicate the sections cut for RNA-seq comparisons. **c** Hematoxylin staining of the scolex and neck, immature, mature and gravid proglottids of *M. expansa*

Parasites from the intestines of freshly slaughtered sheep in an abattoir were immediately transported to the laboratory in a thermally insulated bucket (Thermos®) containing 37 °C phosphate-buffered saline (PBS) (pH 7.4). The collected *Moniezia* were identified as *M. benedeni* or *M. expansa* using hematoxylin staining based on the inter-proglottid glands. The scolex and neck samples included the scolex, neck and a few immature proglottids (total length of *c.*10 mm from apex). The immature samples consisted of *c.*10 mm lengths of tissue 20–30 mm from the scolex. The mature samples consisted of *c.*10 mm lengths of tissue approximately in the middle of the strobila, where both the male and female systems are mature. The fan-shaped vitelline gland was clearly seen by staining the segments. The gravid samples consisted of *c.*10 mm lengths of tissue containing, subterminal tissues in which the internal reproductive organs had degenerated and were full of eggs, confirmed by staining the segments (Fig. 1c).

### Library preparation for transcriptome sequencing

In this study, the four sections, designated the scolex and neck, immature, mature and gravid, and three replicates were used to construct a total of twelve cDNA libraries using the NEBNext® Ultra™ RNA Library Prep Kit for Illumina® (NEB, Ipswich, MA, USA). Briefly, mRNA was purified from the total RNA using poly-T oligo-attached magnetic beads. Fragmentation was carried out using divalent cations under elevated temperature in NEBNext First Strand Synthesis Reaction Buffer (5×). First-strand cDNA was synthesized using random hexamer primer and M-MuLV reverse transcriptase (RNase H-). Second-strand cDNA synthesis was subsequently performed using DNA polymerase I and RNase H. Remaining overhangs were converted into blunt ends *via* exonuclease/polymerase activities. After the adenylation of the 3′ ends of the DNA fragments, NEBNext Adaptor with a hairpin loop structure was ligated to prepare for hybridization. To select cDNA fragments that were preferentially 150–200 bp in length, the library fragments were purified using the AMPure XP system (Beckman Coulter, Beverly, MA, USA). Then, 3 μl of USER enzyme (NEB) was mixed with the size-selected, adaptor-ligated cDNA at 37 °C for 15 min, followed by 5 min at 95 °C, before the PCR. PCR was performed using Phusion High-Fidelity DNA Polymerase, universal PCR primers and Index (X) Primer. Finally, the PCR products were purified (AMPure XP system), and the library quality was assessed using a Bioanalyzer 2100 system (Agilent, Santa Clara, CA, USA).

### Quality analysis, mapping and assembly

The library preparations were sequenced on an Illumina Hiseq 2000 platform, and 100 bp paired-end reads were generated. Then, clean data were obtained by removing reads containing the adapter, reads containing poly-N and low-quality reads from the raw data. In addition, the Q20, Q30 and GC contents of the clean data were calculated. The reference genome and gene model annotation files were unpublished. An index of the *M. expansa* reference genome was built using Bowtie v.2.0.6 [10], and clean reads were aligned to the reference genome using TopHat v.2.0.9 [11]. The mapped reads from each sample were assembled using both Scripture (beta2) and Cufflinks v.2.1.1 [12].

### Gene expression, differential expression, enrichment and co-expression analysis

HTSeq v.0.6.1 [13] was used to count the number of reads mapped to each gene. In addition, the reads per kilobase million (RPKM) of each gene was calculated based on the length of the gene and the number of reads mapped to it. Pairwise differential expression analysis of the four sections defined above was performed using the *DESeq* R package (v.1.10.1) [14]. Genes with an adjusted *P*-value ≤ 0.05 were considered to be differentially expressed. Gene ontology (GO) enrichment analysis of the differentially expressed genes was implemented using the *GOseq* R package [15]. KOBAS software was used to test the statistical enrichment of the DEGs in KEGG pathways [16, 17]. GO terms and KEGG pathways with a corrected *P* ≤ 0.05 were considered to be significantly enriched. Co-expression network analysis was performed using the WGCNA R package [18]. The node and edge information from each module network was input into Cytoscape [19] to visualize and analyze the network modules.

### qRT-PCR verification

The results of the RNA sequencing were validated using qRT-PCR. The total cDNA was synthesized using a reverse transcriptase kit (TaKaRa Biotechnology, Dalian, China). qRT-PCR was performed using a SYBR green assay (TaKaRa Biotechnology) on a Roche LightCycler 480 (Roche Applied Science, Mannheim, Germany). The specific quantitative primers for 8 transcripts are listed in Additional file 1: Table S1. Each 20 µl reaction volume contained 7 µl of H_2_O, 1 µl of each primer, 1 µl of cDNA and 10 µl of 2× Real Master Mix (TaKaRa Biotechnology). The conditions were as follows: an initial single cycle of 95 °C for 3 min; 45 cycles of 95 °C for 15 s, the optimized annealing temperature for 15 s and 72 °C for 20 s; and a final extension step at 72 °C for 5 min. In addition, 10 µl of each PCR product was separated *via* 2% agarose gel electrophoresis. Gene expression levels were normalized to GAPDH to determine the relative expression using the 2^(−ΔΔCt)^ value method. Significant differences in gene expression were analyzed using SPSS v.17.0.

## Results

### RNA sequencing data output summary

An average of 3.05–4.09 G clean reads were generated for the twelve libraries. The GC content of each library was between 40.9–44.17%. In addition, Q20 ≥ 96% and Q30 ≥ 90% indicated that the error rate of a single base was very low. Clean reads were retained and used in the following analysis after discarding reads with adapters or a poly-N content > 10% and other low-quality reads (Additional file 1: Table S2). Finally, 13,874 transcripts were assembled in the 12 libraries.

### Hematoxylin staining results and correlation tests

The correlation coefficients for the three replicates of each of the four body sections were greater than 0.8, indicating that the selected sampling site was effective and that the results were replicable (Fig. 1a). The scolex of *M. expansa* was found to be small and approximately spherical; therefore, both the scolex and neck proglottids were used to obtain sufficient RNA for the library (Fig. 1b). The scolex possesses 4 suckers and lacks rostellum and armature. The immature proglottids showed the early stages of male and female genital primordia, followed by the mature proglottids, which contained mature reproductive organs. *Moniezia expansa* has two sets of reproductive organs; the genital pores are marginal, equatorial; ovary fan-shaped, poral; vitellarium compact, post-ovarian; uterus first reticular, then filling entire proglottids (Fig. 1c).

### qRT-PCR confirmation and differential expression analysis

**Fig. 2:**
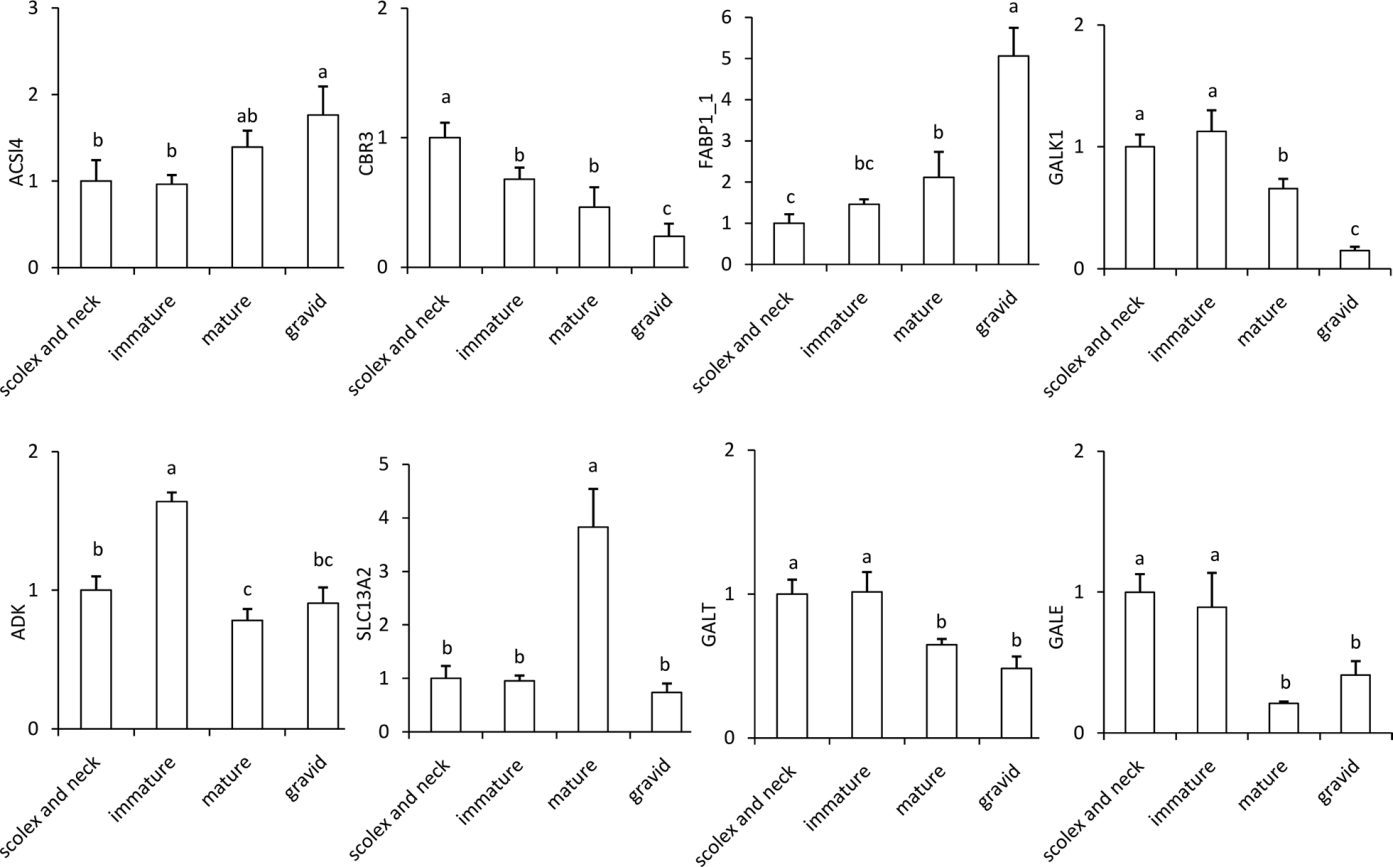
Confirmation of the expression patterns of transcripts using qRT-PCR. Transcript expression was quantified relative to the expression level of GAPDH using the comparative cycle threshold (−ΔΔCt) method. Different superscript letters (a, b, c) indicate significant differences (ASSI4: *F*_(3, 8)_ = 7.91, *P* = 0.009; CBR3: *F*_(3, 8)_ = 22.63, *P* = 10^−9^; FABP1_1: *F*_(3, 8)_ = 44.71, *P* = 10^−9^; GALK1: *F*_(3, 8)_ = 48.87, *P* = 10^−9^; ADK: *F*_(3, 8)_ = 50.41, *P* = 10^−9^; ALC13A2: *F*_(3, 8)_ = 43.78, *P* = 10^−9^; GALT: *F*_(3, 8)_ = 43.78, *P* = 10^−9^; CALE: *F*_(3, 8)_ = 20.51, *P* = 10^−9^)

To verify the RNA-seq results, eight transcripts were selected from the four comparison groups for qRT-PCR, and the primer sequences are shown in Additional file 1: Table S1. The selected transcripts were significantly different in at least one sample comparison. The results show that the expression patterns of these genes were consistent with the RNA-seq results (Fig. 2).

**Fig. 3:**
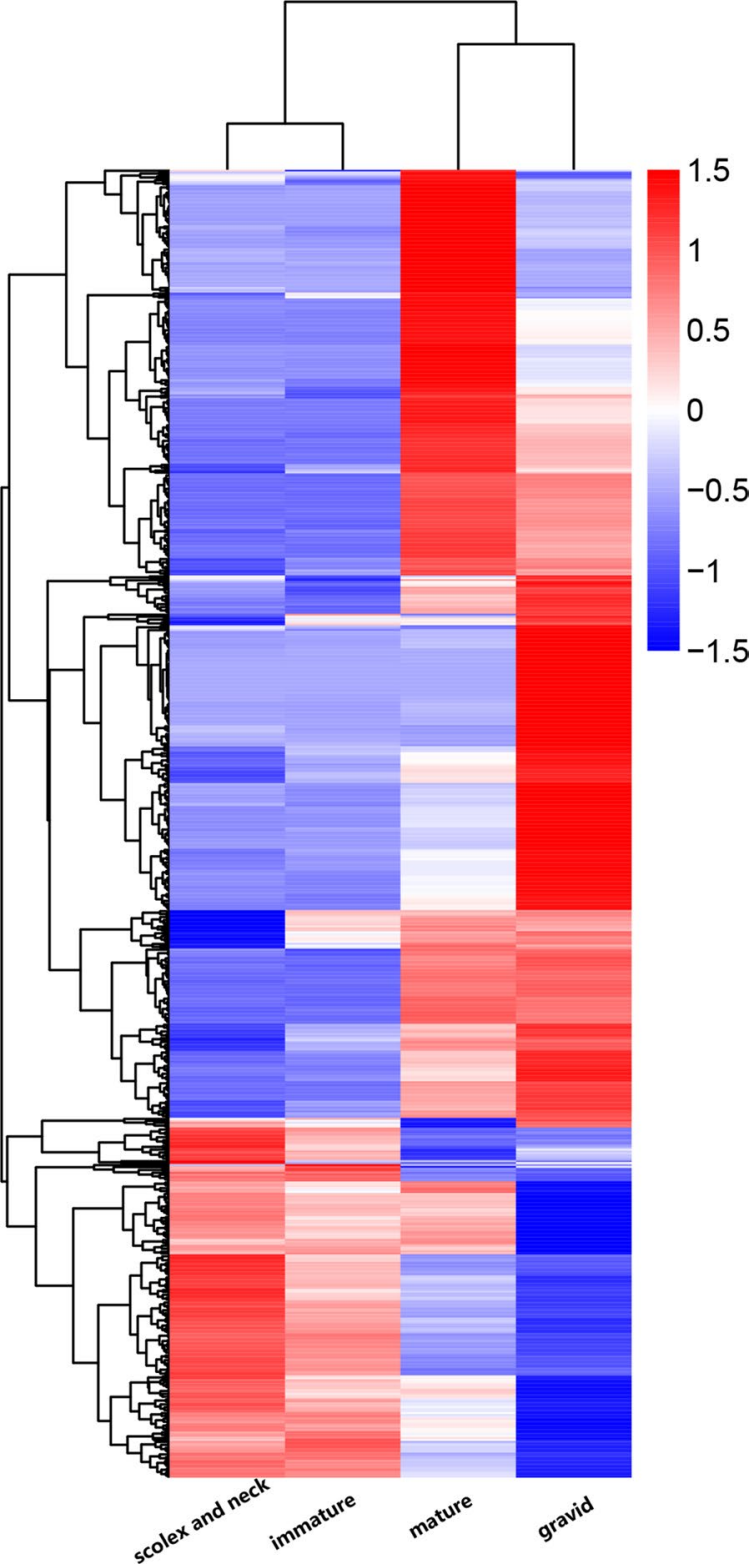
Cluster analysis of DEGs. The overall FPKM hierarchical clustering map was developed using log_10_ (FPKM + 1) values; red indicates high-expression genes, and blue indicates low-expression genes

Using |log2(fold change)| ≥ 1 and *P*-adjusted ≤ 0.05 as cut-offs, 680 differentially expressed genes (DEGs) were obtained from the pairwise comparisons of samples (Table 1). There was only one DEG between the scolex and neck section and the immature proglottids section, likely because the two sampling sites are adjacent. This result can also be seen in the heat map generated based on the DEGs. The gene expression patterns of the scolex and neck and immature proglottids were very similar, while those of the mature and gravid proglottids differed greatly (Fig. 3).

**Table 1:** Numbers of differentially expressed genes in each comparison

Genes	sco-mat	sco-gra	imm-mat	imm-gra	sco-imm	mat-gra
Up	64	136	29	81	1	29
Down	258	256	223	189	0	47
Total	322	392	252	270	1	76

*Abbreviations*: sco, scolex and neck; imm, immature proglottids; mat, mature proglottids; gra, gravid proglottids

### Features associated with high fecundity

Each gravid proglottid of *M. expansa* contains a uterus that is filled with eggs, and it will continuously produce new eggs as these proglottids detach. What kind of substance provides the energy for this high fecundity? In this study, 13 lipid transport-related proteins were found in the DEGs, and the expression level showed an increasing trend in the four proglottid types (Table 2). We hypothesize that *M. expansa* uses lipid transport-associated proteins to transport lipids from the host’s gut to its body, allowing for the production of large numbers of eggs.

**Table 2:** Differentially expressed lipid transport-related proteins

Gene name	BLAST Swiss-Prot	Scolex and neck	Immature	Mature	Gravid
FABP1_1	Fatty acid-binding protein homolog 1	40	231	349	1605
FABP1_2	Fatty acid-binding protein homolog 1	130	226	1052	1574
FABP1_3	Fatty acid-binding protein homolog 1	132	678	665	1756
FABP2_1	Fatty acid-binding protein homolog 2	22	69	336	420
FABP2_2	Fatty acid-binding protein homolog 2	127	238	573	782
SCARB1	Scavenger receptor class B member 1	55	60	144	287
ACSL4	Long-chain-fatty-acid–CoA ligase 4	152	158	189	326
ACSL1	Long-chain-fatty-acid–CoA ligase 1	0	0	19	30
ACACA	Acetyl-CoA carboxylase 1	4	4	5	14
SLC13A2	Solute carrier family 13 member 2	35	28	169	30
SLC22A6-B	Solute carrier family 22 member 6-B	0	0	21	64
SLC10A6	Solute carrier family 10 member 6	5	6	15	16
SLC22A5	Solute carrier family 22 member 5	1	2	38	39

### Homeobox genes

Homeobox genes are highly conserved during evolution and are found within genes that are involved in the regulation of patterns of anatomical development (morphogenesis) in animals [20]. Thirty-three homeobox genes were found in the transcriptome of *M. expansa*, 9 of which were DEGs, and the homeobox genes were most highly expressed in the scolex and neck section. Tapeworms have ganglia and transverse nerves in the scolex, which form the central nervous system; from these, a pair of longitudinal nerve trunks extend to the last proglottids. As such, several of the DEGs were associated with neurodevelopment and exhibited high expression in the scolex and neck section (Table 3).

**Table 3:** Differentially expressed homeobox genes

Homeobox	Gene name	Function
Msx	SLOU	Stimulation of cell proliferation, limb outgrowth, digit elongation and separation [53]
Hox6-8	ARX	Hox genes specify cell identities along the anteroposterior axis of metazoans [54] and play a central role in anterior-posterior patterning, providing a framework for the molecular comparison of animal body plan evolution [55]
	HOXD9	
	ABD-B	
Nk2.1	SMOX-5	Development of the larval anterior neurogenic domains [56]
Irx	SIX6	Irx genes are required for the proper formation of the posterior forebrain, midbrain, and hindbrain and, to a lesser extent, the spinal cord [57]
Meox	Meox1	Meox1 is a novel regulator of TGF-β-induced smooth muscle cell differentiation [58]

### GO and KEGG enrichment analyses

The functional enrichment results for the DEGs were predominantly associated with development-related processes, and there were some signal transduction and metabolism results (Additional file 1: Table S3). The most striking was the Wnt signaling pathways that appeared multiple times. Related studies on flatworms have shown that Wnt signaling pathways can accurately guide regeneration, contribute to stem cell proliferation, regulate the establishment of the anterior-posterior axis (AP axis) and the medial axis, and participate in the formation of the neural system [21]. The Wnt signaling pathway is a complex regulatory network that is currently thought to include three branches: the classical Wnt signaling pathway (the Wnt/β-catenin pathway), the Wnt/planner cell polarity (PCP) pathway and the Wnt/Ca2+ pathway [22]. The DEGs were mainly distributed in the β-catenin and PCP branches (Table 4), such as the Fzd gene in the classical Wnt signaling pathway, secreted glycoprotein, 7 transmembrane proteins that are similar in structure to G protein-coupled receptors and can bind Wnt, and casein kinase (CK), which can phosphorylate the Ser45 site of β-catenin.

**Table 4:** Enrichment genes of the Wnt signaling pathway

Gene name	Wnt branches	BLAST Swiss-Prot
FZD10	All	Frizzled-10
FZD5	All	Frizzled-5
SFRP5	All	Secreted frizzled-related protein 5
WNT11B	PCP	Protein Wnt-11b
WNT11	PCP	Protein Wnt-11
SCON-3_1	β-catenin	E3 ubiquitin ligase complex SCF subunit scon-3
SCON-3_2	β-catenin	E3 ubiquitin ligase complex SCF subunit scon-3
ASK4	β-catenin	SKP1-like protein 4
CSNK2A1	β-catenin	Casein kinase II subunit alpha (*Mus musculus*)
CKA1	β-catenin	Casein kinase II subunit alpha (*Schizosaccharomyces pombe*)
CKA2	β-catenin	Casein kinase II subunit alpha (*Saccharomyces cerevisiae*)
CKA	β-catenin	Casein kinase II subunit alpha (*Neurospora crassa*)
EP300	β-catenin	Histone acetyltransferase p300
WNT2B-A	β-catenin	Protein Wnt-2b-A

### WGCNA

**Fig. 4:**
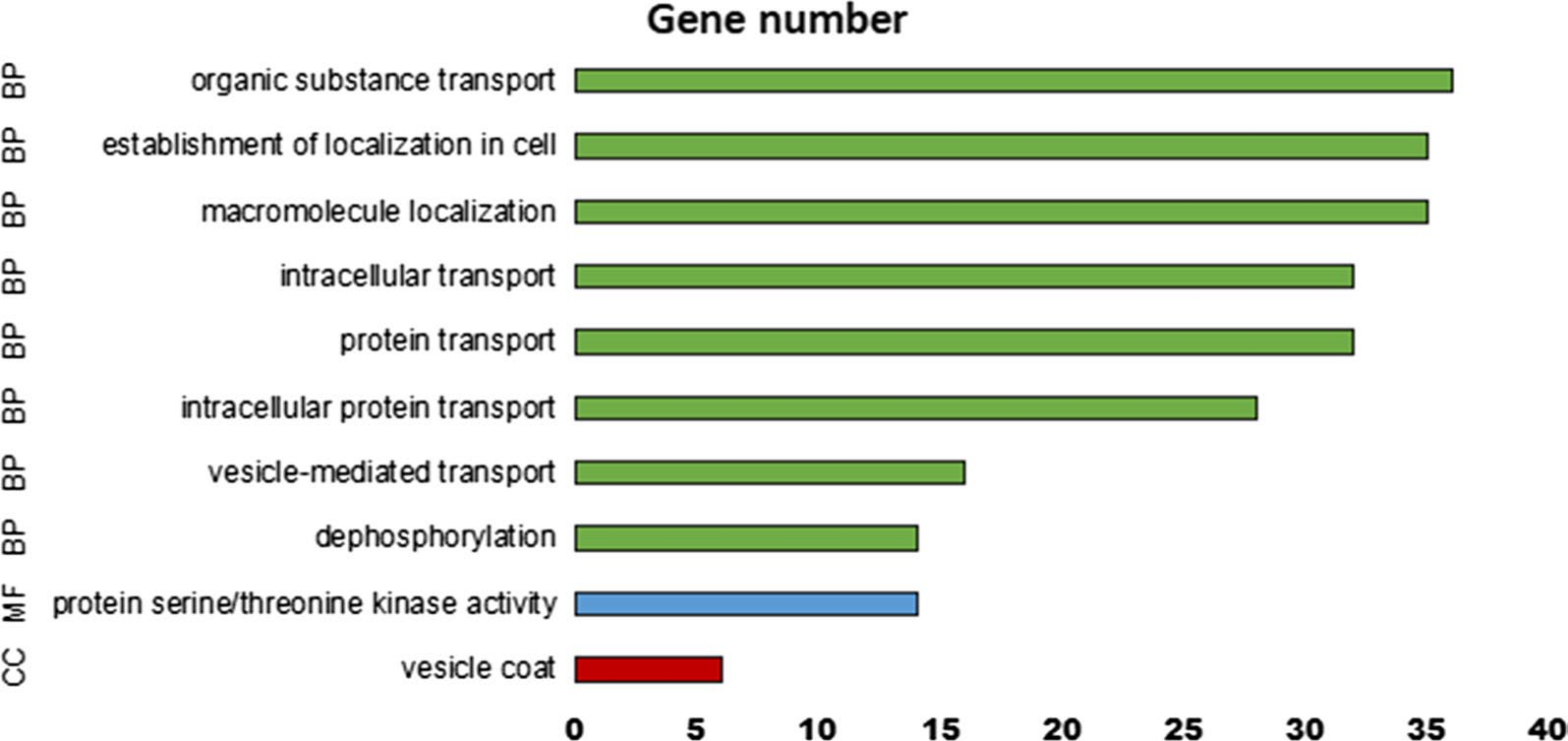
GO enrichment plot of the DEGs in the brown module. *Abbreviations*: BP, biological process; CC, cell composition; MF, molecular function

**Fig. 5:**
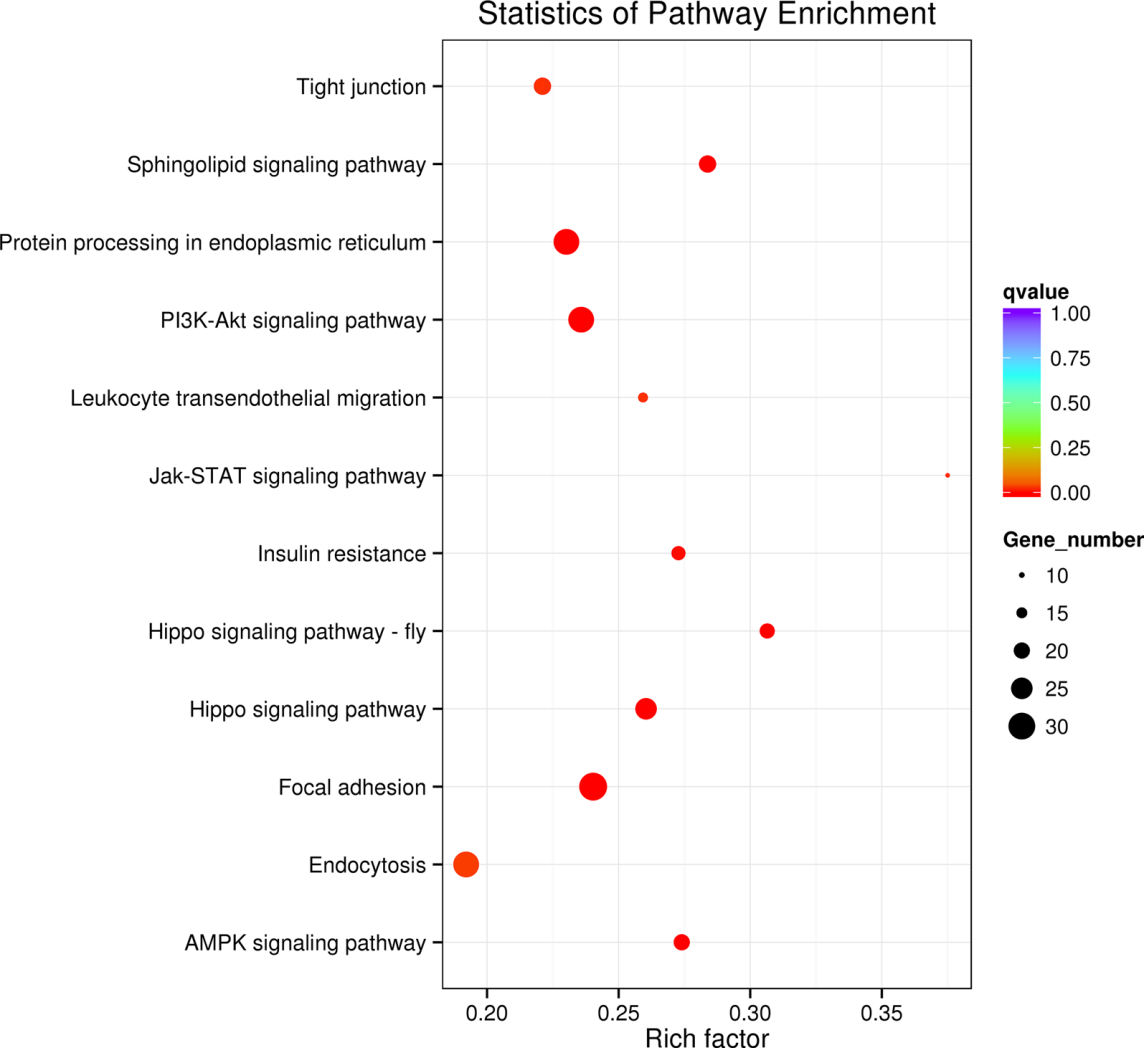
KEGG enrichment scatter plot of DEGs in the brown module. *Note*: Rich factor refers to the ratio of the number of genes located in the pathway entry in the DEGs to the total number of genes in the annotated genes located in the pathway entry. The greater the rich factor, the greater the degree of enrichment. The q-value indicates the *P*-value after correction for the testing of multiple hypotheses. The range of the q-value is [0, 1], with values closer to zero indicating more significant enrichment

WGCNA is an analytical method used for complex samples that mines module information from sequencing data. In this method, a module is defined as a group of genes with similar expression profiles. If some genes always undergo similar expression changes during a physiological process or in different tissues, then there is reason to believe that these genes are functionally related and are thus defined as a module. The expression values of the transcripts of the 12 samples were considered and a total of 12 modules were constructed using different colors in the WGCNA network (Additional file 1: Figure S1). The squares along the heat map network diagonal represent modules that were darker than the adjacent squares (Additional file 1: Figure S2), and the genes within the modules exhibited more topological overlap than the genes across the modules. The brown module was found to be enriched with many development-related genes through the functional annotation of the genes in the modules. The GO term, which is related to the transport of proteins and organic substances, showed enrichment (Fig. 4), along with the pathways related to development (hippo signaling pathway and hippo signaling pathway—fly), signal transduction (PI3K-Akt, AMPK, sphingolipid signaling pathway and Jak-STAT signaling pathways), cellular community (tight junction and focal adhesion), metabolic diseases (insulin resistance) and transport (endocytosis) (Fig. 5).

### Co-expression network of the genes in the brown module

**Fig. 6:**
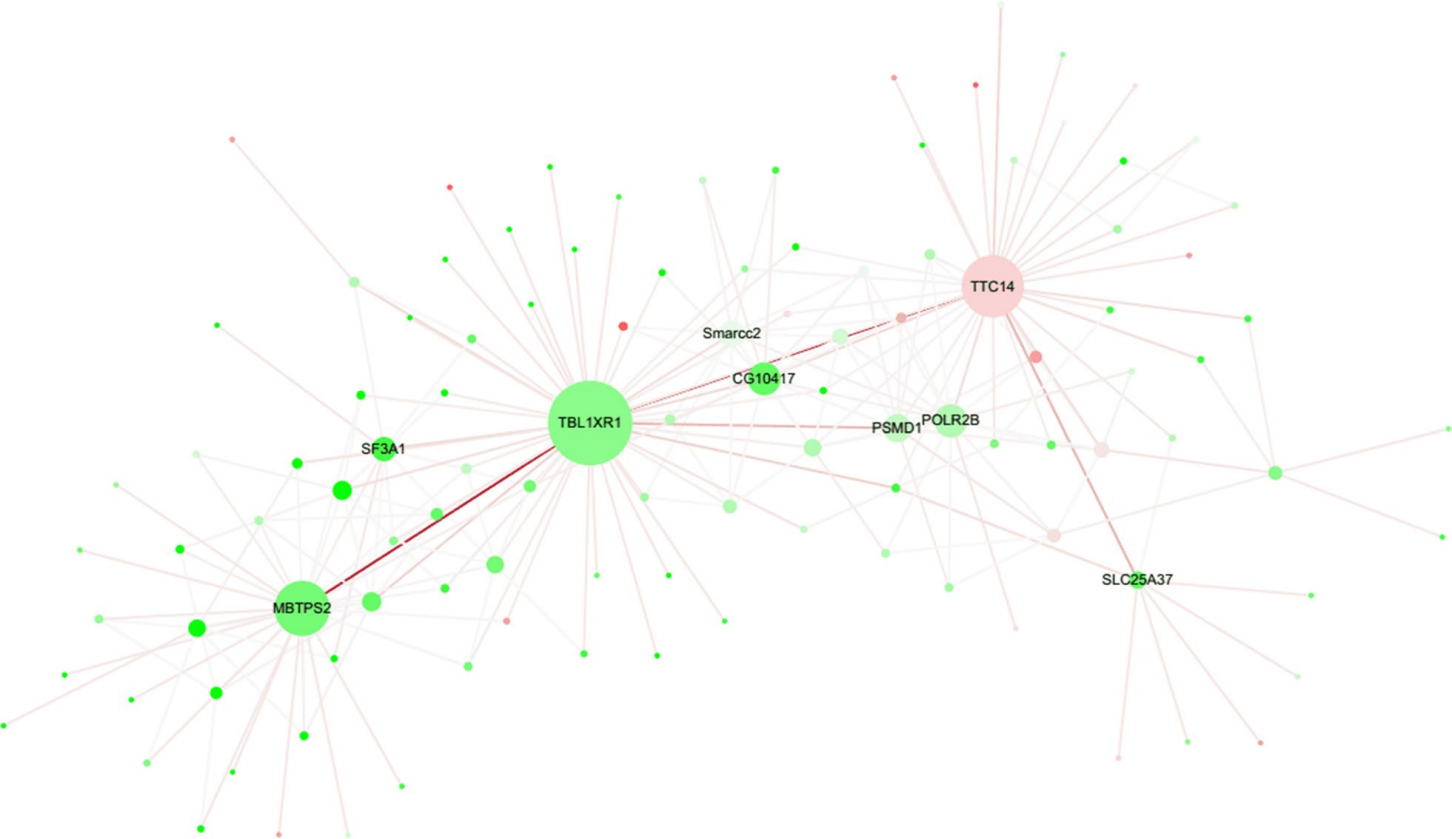
Co-expression network of the genes in the brown module. Node color denotes differential expression levels. Green represents downregulation, and red represents upregulation. Node size represents the importance of a node. Edge denotes the interaction strength

Core genes in the co-expression network associated with binding, activation and transport, which were divided into three categories, were revealed by the WGCNA of the genes in the brown module (Fig. 6). In the first category, the molecular function of TTC14 (tetratricopeptide repeat protein 14) is protein-binding, that of POLR2B (DNA-directed RNA polymerase II subunit RPB2) is DNA-binding, and SMARCC2 (SWI/SNF related-matrix-associated actin-dependent regulator of chromatin subfamily C and member 2) is associated with chromatin-binding. In the second category, MBTPS2 (membrane-bound transcription factor site-2 protease) regulates the activity of metalloendopeptidase, and CG10417 (protein phosphatase 2C) has catalytic activity. In the third category, SLC25A37 (solute carrier family 25, member 37) is a mitochondrial iron transporter, and TBL1XR1 (transducin (beta)-like 1) is one of the G protein-coupled receptor (GPCR) family members.

## Discussion

Parasites obtain all of their nutrients from their hosts and are also greatly affected by the body fluid environment in which they live. For instance, female *Schistosoma haematobium* living in the blood vessels exhibits major enrichment in pathways linked to hematophagy (including those related to superoxide dismutase, saposin, cathepsin B and ferritin) and egg production (including those related to lipid metabolism, protein synthesis and eggshell-specific proteins) [23]. In addition, *Opisthorchis viverrini* living in the bile-duct transcribes a series of enzymes to degrade lipoprotein complexes and transport free amino acids derived from the bile to the body *via* amino acid transporters [24]. *Moniezia expansa* lives in the fluidin the small intestine, which has the primaryrole in the absorption of nutrients. A large number of differentially expressed fatty acid transporters were found in *M. expansa* (Table 2); among them, the main function of fatty acid-binding proteins (FABPs) is to transport fatty acids, especially polyunsaturated fatty acids. The FABP family is divided into four broad categories: the first category consists of vitamin A derivative-specific-binding proteins, including intracellular retinoid-binding proteins (CRABPI and CRABPII) and intracellular retinol-binding proteins (CRBPI, CRBPII, CRBPIII and CRBPIV); the second type of FABPs generally bind to larger ligands, such as bile acids, hemes, eicosanoids, etc. including I-LBP (ileum), L-FABP (liver) and Lb-FABP (liver basic); the third type of FABPs has only one member I-FABP (intestinal); and the fourth type of FABPs includes H-FABP (heart), AFABP (adipocyte), E-FABP (epidermal), M-FABP (myelin), T-FABP (testis), and B-FABP (brain) [25]. The fatty acid-binding protein identified in *M. expansa* was mostly I-FABP (FABP2) (small intestinal type), which is closely related to its environment in the small intestine fluid. This finding explains why *M. expansa* has such high levels of body fat in the absence of lipid synthesis: it uses FABPs to transport lipids in the host intestinal fluid to the body. In addition, the brown module interaction network map showed that SLC25A37 is a centrally located key gene (Fig. 4). Studies have shown that genetic polymorphisms in solute carriers affect the selectivity of fatty acid intake [26]. All evidence indicates that *M. expansa* uses lipids for energy to facilitate its continuous reproduction and development.

As the most primitive metazoan phylum, the Platyhelminthes occupies a unique position in nervous system evolution [27]. The nervous system appears to be a modified ladder-type, with a longitudinal cord near each lateral margin and transverse commissures in each proglottid. The two lateral cords are united in the scolex in a complex arrangement of ganglia and commissures [28]. In *M. expansa*, the longitudinal nerve cord is reduced, with only one remaining pair of ventral nerve cords running through all of the proglottids that form trapezoidal transverse nerve connections with various parts of the body. Only seven homeobox genes have been found to be related to neural development in *M. expansa* (Gbx [29], Gsx [30], Lbx [31], Rax [32], Prrx [33], Pou4 [34], Pou6 [35] and Nk2.1 [33]), whereas other neural development-related homeobox genes (Mnx, Pax3-/-7, Hbn, Evx, Dlx, Onecut, Prox, Adnp, Tshz, Otp, Otx, Phox, Uncx, Lhx3/4, Pou1 and Hesx) have not been found. These results are consistent with the fact that *M. expansa* has an underdeveloped nervous system. In particular, to our knowledge, this is the first time that Gbx, Hbn and Rax have been found in a tapeworm. Previous studies have shown that those homeobox genes are only present in the trematodes (*Schistosoma mansoni* and *Schistosoma japonicum*), and are missing in the tapeworms (*Echinococcus multilocularis*, *Echinococcus granulosus*, *Taenia solium* and *Hymenolepis microstoma*) [36]. Specifically, many senses in tapeworms (visual, gustatory, tactile and auditory) have been degraded due to their parasitic lifestyle. Compared with other species, many homeobox genes have been lost in *M. expansa*, including 297 of the sequences in humans, 344 of the sequences in zebrafish, 107 of the sequences in *Drosophila* and 99 of the sequences in *C. elegans*. These lost homeobox genes are inseparable from the parasitic lifestyle of *M. expansa*; for example, CDX, which is associated with intestinal mucosa and gastric mucosal development; Hox5, which plays a role in lung morphogenesis [37]; Hox9-13, which are expressed during kidney development [38]; Pdx, which is the main control gene in the pancreas [39]; Hhex, which functions as the transcriptional regulator during vascular development [40]; Six1/2, which is related to eye development [41]; and Prop, which is associated with the phenotypic expression of taste [42].

Planarians have the ability to regenerate; regardless of whether they are cut along the sagittal, cross-section or frontal planes, the cut parts can regenerate new, intact individuals at the original scale. Indeed, the stem cells of planarians have the ability to regenerate a head or tail. When Wnt/β-catenin signaling is enhanced, planarians gradually develop toward the back end, which causes the regeneration of the tail structure [43]; in contrast, when Wnt/β-catenin signaling is weakened, development toward the front end causes the regeneration of the front head structure [36]. The discovery that Wnt/β-catenin signaling is responsible for regulating head/tail specification in planarian regeneration has recently highlighted its importance in flatworm development. Furthermore, many members of the Wnt signaling pathway have also been reported in cestodes. For instance, *Echinococcus multilocularis*, *Echinococcus granulosus*, *Hymenolepis microstoma* and *Taenia solium* all contain Wnt1, Wnt2, Wnt4, Wnt5, Wnt11a, Wnt11b, FrizzledA, FrizzledB, FrizzledC, FrizzledD, FrizzledE, Dishevelled, GSK3, APC, Axin, β-catenin A, β-catenin B and LEF1/TCF [44]. In this study, FZD10, FZD5, SFRP5, WNT11B, WNT11, SCON-3, ASK4, CSNK2A1, CKA1, EP300 and WNT2B-Awere characterized in *M. expansa*. The overall expression level of these genes was highest in the scolex and neck section, with the highest expression levels occurring for the Frizzled proteins and WNT11. This result is consistent with previous studies of the highly derived larval form of *E. multilocularis*, which proliferates asexually within mammalian hosts; this species demonstrates posterior expression of specific Wnt factors during larval metamorphosis, and scolex formation is preceded by the localized expression of Wnt inhibitors [45]. Furthermore, Wnt signaling activators (positive regulatory genes) such as Wnt1, Wnt11-1, Wnt11-2, Wnt11-5 and frz-4, are mainly expressed at the posterior end of the worm [46], whereas sfrp-1 and the Wnt inhibitor notum are mainly expressed at the front end [47]. However, RNAi treatment of Smed-β-catenin-1, Smed-wnt1, Smed-wnt2, Smed-wnt11-2, Smed-evi/wntless, Smed-teashirt (Tsh) and Smed-dishevelled-1/2 of the Wnt signaling pathway resulted in abnormal development of the tail and regeneration of the head of planarians [48], while RNAi treatment of the Smed-notum, Smed-axin and APC genes caused abnormal development of head and regenerated the tail [49]. The strong conservation of gene expression in tapeworms and planarians suggests a homologous developmental period across this diverse phylum. Such homologous developmental periods are postulated to represent the phylotypic stages of these flatworm taxa. The results support the classical notion that segmentation in *M. expansa* (and in cestodes in general) is induced anteriorly from the scolex and neck region and that the Wnt signaling pathway plays the core role in this process.

Notably, the differentially expressed genes between the scolex and neck and mature sections were enriched in signaling pathways regulating the pluripotency of stem cells, among which there were three very interesting genes. The first was BMP2 (bone morphogenetic protein 2). Studies have shown that BMP2 promotes the calcification of cystic wall cells in hydatid cysts. Tapeworms will experience decreased survival when the cyst wall is calcified because a high level of cyst wall calcification makes it difficult for the worm to take up nutrients from the surrounding tissue [50]. The second is FGFR1 (fibroblast growth factor receptor 1). In recent years, an increasing number of studies have confirmed that fibroblasts have the characteristics of stem cells and exhibit the potential for multidirectional differentiation. Under the intervention of *in vitro* induction, they can differentiate into lipids, bone and heart muscle [51]. Finally, Myf5 (myogenic factor 5), which is very important in the process of muscle formation, is related to the number and size of muscle fibers [52]. The high expression of these genes in scolex and neck section seems to confirm that components of the neck sections have stem cell characteristics.

## Conclusions

This study used both RNA sequencing and WGCNA to analyze the expression of transcripts in the different cestode regions (scolex and neck, immature, mature and gravid proglottids) of *M. expansa*. The dynamic expression profiles of the developmental and molecular basis of the high body fat percentage in this species were elucidated, and the regulatory role of transcripts in development was explored based on the Wnt signaling pathway and homeobox genes. The results provide an explanation for the high body fat percentage and continuous regeneration properties of *M. expansa*.

## Additional file

**Additional file 1: Table S1.** Primer sequences of the 12 transcripts selected for qRT-PCR. **Table S2.** Sequencing data quality summary. **Table S3.** GO and KEGG enrichment results for the differentially expressed genes. **Figure S1.** Relationships between the modules and samples. **Figure S2.** Topological overlap heat map of the gene co-expression network.

## Data Availability

The datasets generated during the present study are available in the Sequence Read Archive repository (SRA: PRJNA542191), https://dataview.ncbi.nlm.nih.gov/object/PRJNA542191

## Abbreviations

DEG: differentially expressed gene
WGCNA: weighted gene co-expression networks
qRT-PCR: real-time polymerase chain reaction
GAPDH: glyceraldehyde-3-phosphate dehydrogenase
FPKM: fragments per kilobase million
PBS: phosphate-buffered saline
