# Supplementary material for: Evaluation of dynamic developmental processes and the molecular basis of the high body fat percentage of different proglottid types of Moniezia expansa
Source: Parasit Vectors. 2019 Aug 5;12:390. doi: 10.1186/s13071-019-3650-1 (PMC6683355; doi:10.1186/s13071-019-3650-1)
Supplement: Supplementary file 1 — Additional file 1: Table S1. Primer sequences of the 12 transcripts selected for qRT-PCR. Table S2. Sequencing data quality summary. Table S3. GO and KEGG enrichment results for the differentially expressed genes. Figure S1. Relationships between the modules and samples. Figure S2. Topological overlap heat map of the gene co-expression network. [file 13071_2019_3650_MOESM1_ESM.docx]

**Additional file 1: Table S1.** Primer sequences of the 12 transcripts selected for qRT-PCR.

| **Transcript id** | **Primer sequence (5'→3')** | **Amplicon length (bp)** |
| --- | --- | --- |
| SLC13A2 | F:CGGGCTCTGAAGGGAAA | 235 |
|  | R:CTAATACGGCGGTGGATGT |  |
| FABP1_1 | F:GTAGTGAGGGATTCGACAAGG | 170 |
|  | R:ACCCAAACGGAAGCAGG |  |
| ADK | F:AGCGGTAAGGGCACTCAA | 277 |
|  | R:GGGCAACCTCTTTTTCAAA |  |
| ACSl4 | F:GAGGGGGGATGAGGGTGTTA | 175 |
|  | R:GTTGTGTGGATTGAAGTGCG |  |
| CBR3 | F:TTTGGGAAAGGAGGCTGT | 125 |
|  | R:CGGGGTAGTTCTGTTTAGTGA |  |
| GALK1 | F:TGCTTGTCTCGGAGGTGTT | 293 |
|  | R:GCAGTCTTTTAGTGTGGGCT |  |
| GALE | F:CATTTTCCCCTGTCTTACGA | 351 |
|  | R:TTCTCCGCTTTCCACCC |  |
| GALT | F:GAGGCAGCCACTGAGAAAG | 337 |
|  | R:TGGGATCAACAGCAAACAA |  |

**Additional file 1: Table S2.** Sequencing data quality summary.

| **Sample** | **Raw reads** | **Clean reads** | **Clean bases** | **Q20 (%)** | **Q30 (%)** | **GC (%)** |
| --- | --- | --- | --- | --- | --- | --- |
| Scolex and neck_a_1 | 24459952 | 23826543 | 3.57G | 97.4 | 92.97 | 42.22 |
| scolex and neck_a_2 | 24459952 | 23826543 | 3.57G | 96.32 | 90.63 | 42.31 |
| scolex and neck_b_1 | 25148916 | 24766508 | 3.71G | 97.73 | 93.7 | 42.36 |
| scolex and neck_b_2 | 25148916 | 24766508 | 3.71G | 97.21 | 92.17 | 42.34 |
| scolex and neck_c_1 | 23159764 | 22914660 | 3.44G | 97.86 | 93.98 | 42.38 |
| scolex and neck_c_2 | 23159764 | 22914660 | 3.44G | 97.3 | 92.35 | 42.35 |
| immature_a_1 | 23825184 | 23276628 | 3.49G | 97.38 | 92.96 | 43.08 |
| immature_a_2 | 23825184 | 23276628 | 3.49G | 95.05 | 88.04 | 43.14 |
| immature_b_1 | 26005701 | 25731139 | 3.86G | 97.78 | 93.84 | 44.26 |
| immature_b_2 | 26005701 | 25731139 | 3.86G | 96.92 | 91.57 | 44.29 |
| immature_c_1 | 22066629 | 21797818 | 3.27G | 97.85 | 93.94 | 42.83 |
| immature_c_2 | 22066629 | 21797818 | 3.27G | 97.56 | 92.97 | 42.84 |
| mature_a_1 | 20743673 | 20360170 | 3.05G | 97.46 | 93.05 | 40.68 |
| mature_a_2 | 20743673 | 20360170 | 3.05G | 95.64 | 89.11 | 40.7 |
| mature_b_1 | 25677893 | 25344795 | 3.8G | 97.65 | 93.54 | 40.91 |
| mature_b_2 | 25677893 | 25344795 | 3.8G | 97 | 91.71 | 40.9 |
| mature_c_1 | 24300346 | 23869165 | 3.58G | 97.79 | 93.97 | 43.66 |
| mature_c_2 | 24300346 | 23869165 | 3.58G | 97.43 | 92.83 | 43.65 |
| gravid_a_1 | 25331359 | 24721001 | 3.71G | 97.47 | 93.08 | 41.43 |
| gravid_a_2 | 25331359 | 24721001 | 3.71G | 95.95 | 89.69 | 41.44 |
| gravid_b_1 | 27656788 | 27254218 | 4.09G | 97.75 | 93.91 | 44.13 |
| gravid_b_2 | 27656788 | 27254218 | 4.09G | 96.6 | 90.91 | 44.11 |
| gravid_c_1 | 22551667 | 22270913 | 3.34G | 97.6 | 93.67 | 43.28 |
| gravid_c_2 | 22551667 | 22270913 | 3.34G | 96.49 | 90.84 | 43.25 |

**Additional file 1: Table S3.** GO and KEGG enrichment results for the differentially expressed genes.

| **mature vs gravid. KEGG. result** | |
| --- | --- |
| map05218 | Melanoma |
| **immature vs mature. KEGG. result** | |
| map04712 | Circadian rhythm - plant |
| map04310 | Wnt signaling pathway |
| map04064 | NF-kappa B signaling pathway |
| map05168 | Herpes simplex infection |
| map04139 | Regulation of mitophagy - yeast |
| **immature vs gravid. KEGG. result** | |
| map00052 | Galactose metabolism |
| map00511 | Other glycan degradation |
| **scolex and neck vs mature. KEGG. result** | |
| map04310 | Wnt signaling pathway |
| map04712 | Circadian rhythm - plant |
| map04550 | Signaling pathways regulating the pluripotency of stem cells |
| map05168 | Herpes simplex infection |
| map04064 | NF-kappa B signaling pathway |
| **scolex and neck vs gravid. GO. result** | |
| GO:0044767 | Single-organism developmental process |
| GO:0007156 | Homophilic cell adhesion |
| GO:0005102 | Receptor binding |
| GO:0007275 | Multicellular organismal development |
| GO:0048523 | Negative regulation of cellular process |
| GO:0016055 | Wnt signaling pathway |
| GO:0005509 | Calcium ion binding |
| GO:0030154 | Cell differentiation |
| **immature vs mature. GO. result** | |
| GO:0004887 | Thyroid hormone receptor activity |
| GO:0006355 | Regulation of transcription, DNA-templated |
| GO:0004252 | Serine-type endopeptidase activity |
| GO:0034654 | Nucleobase-containing compound biosynthetic process |
| GO:0008152 | Metabolic process |
| GO:0006508 | Proteolysis |
| GO:0043170 | Macromolecule metabolic process |
| GO:0071704 | Organic substance metabolic process |
| GO:0044238 | Primary metabolic process |
| **immature vs gravid. GO. result** | |
| GO:0006012 | Galactose metabolic process |
| GO:0019318 | Hexose metabolic process |
| GO:0004553 | Hydrolase activity, hydrolyzing O-glycosyl compounds |
| **scolex and neck vs mature. GO. result** | |
| GO:0006355 | Regulation of transcription, DNA-templated |
| GO:0044767 | Single-organism developmental process |
| GO:0007275 | Multicellular organismal development |
| GO:0016055 | Wnt signaling pathway |
| GO:0004887 | Thyroid hormone receptor activity |
| GO:0048513 | Organ development |
| GO:0005102 | Receptor binding |
| GO:0034654 | Nucleobase-containing compound biosynthetic process |
| GO:0006508 | Proteolysis |
| GO:0042813 | Wnt-activated receptor activity |
| GO:0004252 | Serine-type endopeptidase activity |
| GO:0043565 | Sequence-specific DNA binding |


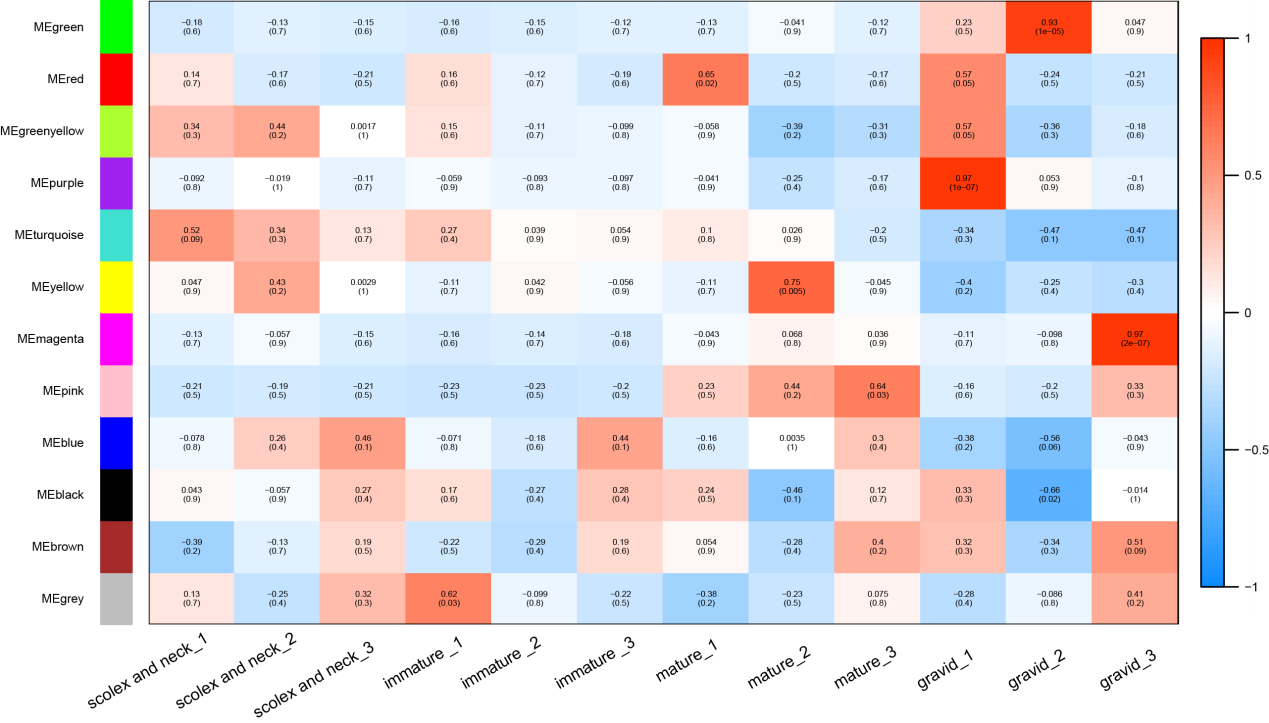


**Additional file 1: Figure S1.** Relationships between the modules and samples. Hierarchical clustering dendrogram of the module eigengenes and a heat map of the adjacencies using weighted coexpression network analysis. Blue represents a negative correlation, and red represents a positive correlation.


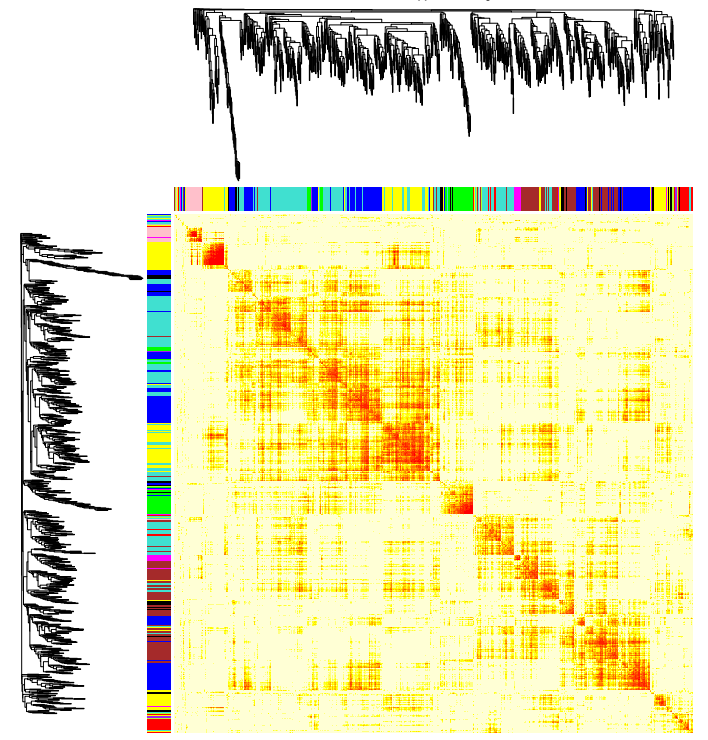


**Additional file 1: Figure S2.** Topological overlap heat map of the gene coexpression network. Each row and column represents a gene. A light color indicates low topological overlap, and progressively darker colors indicate increased topological overlap. Darker squares along the diagonal represent modules. The gene dendrogram and module assignment are displayed along the left and top of the figure.
